# Optimization of Perovskite Tandem Photovoltaic Devices for Terrestrial and Space-Based Applications Using External Quantum Efficiency Simulations

**DOI:** 10.3390/ma19173618

**Published:** 2026-08-26

**Authors:** Emily Amonette, Nikolas J. Podraza

**Affiliations:** Wright Center for Photovoltaics Innovation and Commercialization, University of Toledo, Toledo, OH 43606, USA; emily.miller12@utoledo.edu

**Keywords:** photovoltaics, perovskite, photovoltaic simulation, tandem photovoltaics

## Abstract

The absorber layer thicknesses of tandem photovoltaic devices containing hybrid organic–inorganic lead halide perovskite absorbers are optimized under AM 1.5 and AM 0 solar irradiance using external quantum efficiency (EQE) simulations. Using the EQE modeling approach derived from analysis of ellipsometric spectra collected from complete single-junction perovskite, all-perovskite tandem, and copper indium gallium diselenide (CIGS) thin film solar cells, structural–optical models are developed for two high-efficiency tandem solar cell configurations from their published EQE spectra. These configurations include a superstrate all-perovskite device and a substrate perovskite/CIGS device. These models serve as realistic and practical baselines for optimizing device performance under different circumstances. By increasing the thicknesses of an all-perovskite tandem superstrate device’s wide E_g_ and narrow E_g_ absorber layers from 350 and 975 nm to 356 and 1200 nm, the J_sc_ may be increased from 15.81 to 15.94 mA/cm^2^ under AM 1.5 illumination. This corresponds to a potential increase in efficiency from 25.83 to 26.05% when using reported open circuit voltage (V_oc_) and fill factor (FF). Under AM 0, an increase in absorber layer thickness to 310 and 1200 nm increases the J_sc_ from 18.56 to 19.72 mA/cm^2^, which corresponds to an increase in efficiency from 30.33 to 32.22%. By increasing the thickness of the perovskite layer in a perovskite/CIGS substrate device from 500 to 615 nm, the J_sc_ may be increased from 18.84 to 19.65 mA/cm^2^ assuming AM 1.5 illumination. This change would increase efficiency from 23.74 to 24.76%. Under AM 0 illumination, an increase in the perovskite thickness to 512 nm results in an increase in predicted J_sc_ from 23.13 to 23.34 mA/cm^2^. This corresponds to a predicted efficiency increase from 29.15 to 29.41%. This modeling approach provides a stable platform for practical evaluation of different superstrate and substrate design tandem solar cells with perovskite semiconductors as at least one of their absorber layers.

## 1. Introduction

Tandem photovoltaic (PV) devices offer the potential of greatly increased efficiencies compared to single-junction PV devices. Hybrid organic–inorganic lead halide-based perovskites and copper indium gallium diselenide (CIGS) semiconducting absorber layers feature tunable band gap (E_g_) energies that vary with composition, making them promising absorber layer materials for wide and narrow E_g_ subcells in tandem PV devices and other optoelectronic devices [1]. Current record efficiencies of tandem devices containing perovskite absorbers are 30.1% for all-perovskite devices, 34.6% for perovskite/Si devices, and 24.6% for perovskite/CIGS devices [2]. Researchers constantly explore excursions in process parameter space such as layer thicknesses to increase efficiency, and in the case of tandem devices, the number of adjustable parameters can be numerous. By simulating the external quantum efficiency (EQE) spectra of tandem devices, the effects of parameter adjustments on short circuit current density (J_sc_) can be readily determined. This would be helpful in aiding experimentalists in improving their devices. This work first focuses on how to develop physically realistic optical–structural models for a superstrate design in an all-perovskite tandem device and a substrate design in a perovskite/CIGS tandem device from reported experimental EQE. This capability enables a better framework for establishing practical limitations relative to simulated device performance. The simulated all-perovskite device is based on the work of Wen et al. at Nanjing University, which held the record for all-perovskite tandem device efficiency of 26.4% in 2021 [2,3]. The simulated perovskite/CIGS device is based on the work of Jošt et al. at the Helmholtz Center Berlin for Materials and Energy, which held the record for perovskite/CIGS tandem device efficiency of 24.2% from February 2020—January 2025 [2,4]. Baseline structural–optical models for these devices are developed from analysis of their EQE spectra, building from our previous work in modeling perovskite and CIGS solar cell EQE using input parameters from spectroscopic ellipsometry [5,6,7,8,9,10,11]. This ability enables the generation of a realistic optical–structural model from reported EQE spectra which in turn can be used to identify future J_sc_ optimization pathways. As many solar cell devices are optimized for terrestrial applications at AM 1.5 solar illumination when reported in the literature, this approach can be applied to identify how current matching in tandem devices can be achieved under AM 0 solar illumination for space applications.

## 2. Methods

EQE simulations are performed using free, publicly available software (e-ARC 3.0) that uses a transfer matrix approach where reflectance and absorbance spectra of each layer are calculated [9]. The thicknesses of the absorber layers are divided into multiple sections, and the contribution to the EQE of some sections are reduced until the simulated EQE spectra match the experimental EQE spectra, simulating the effect of electronic losses [8]. The resulting spectra are used to calculate J_sc_. The J_sc_ of each absorber layer and the equivalent J_sc_ loss of all other layers are calculated by integrating the absorbance spectra multiplied by the photon energy and solar irradiance spectrum with respect to wavelength [8]. J_sc_ losses due to reflectance and electronic losses are similarly calculated using their respective spectra. Irradiance of each layer is calculated by integrating the absorbance spectra multiplied by the solar irradiance spectrum [10].

For the all-perovskite superstrate device, planar interfaces are assumed at all wavelengths. For the CIGS/perovskite substrate device, planar interfaces are assumed at wavelengths less than 750 nm, and minimized reflection loss due to scattering is assumed at wavelengths greater than or equal to 750 nm. This is done to better approximate the shape of the experimental EQE spectra. Polycrystalline perovskite and CIGS thin films often exhibit scattering at long wavelengths comparable to their grain size [6,11]. Complex dielectric function spectra and thicknesses of each layer are input parameters.

**Figure 1:**
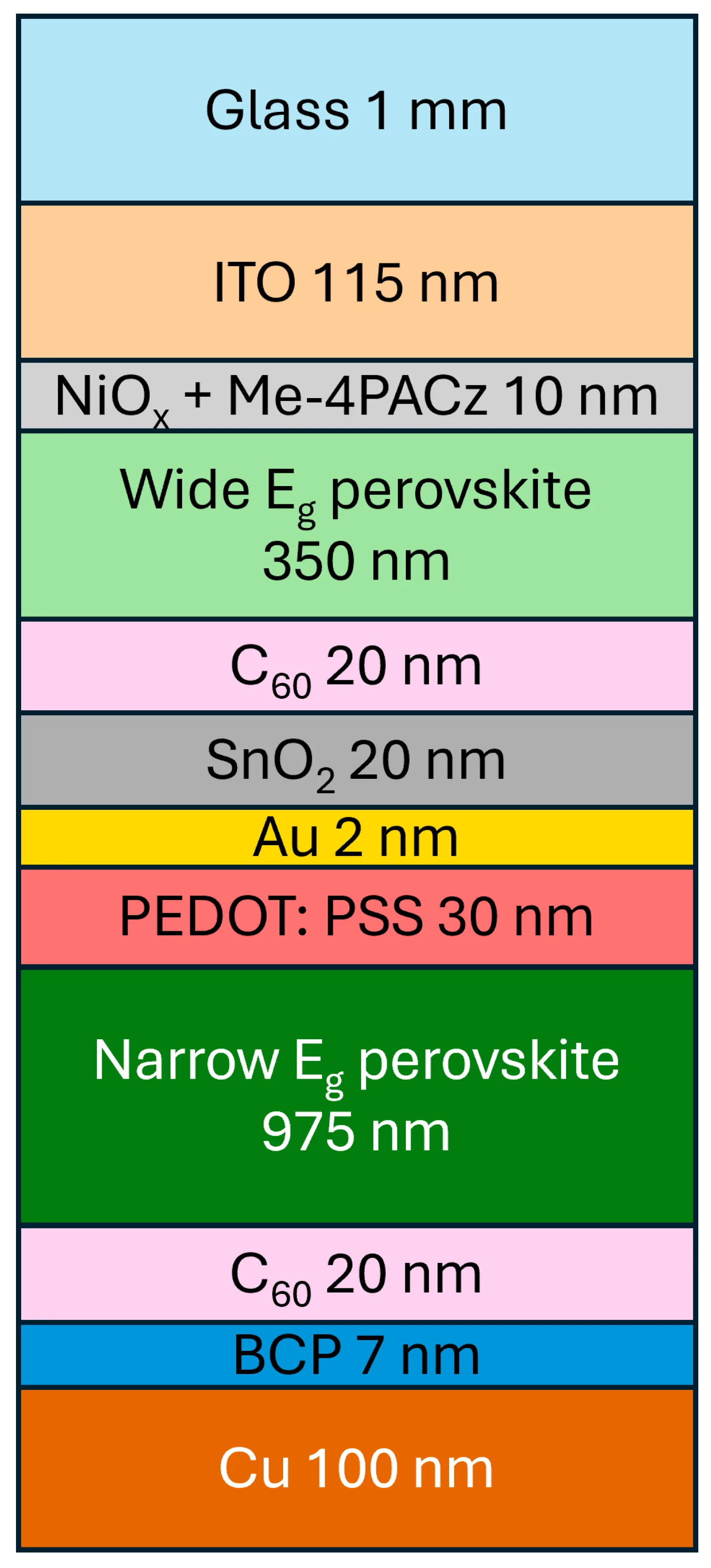
Nominal structural model used to simulate external quantum efficiency (EQE) spectra of an all-perovskite tandem superstrate photovoltaic (PV) device. Thicknesses are chosen to approximate the EQE published by Wen et al. [3].

**Figure 2:**
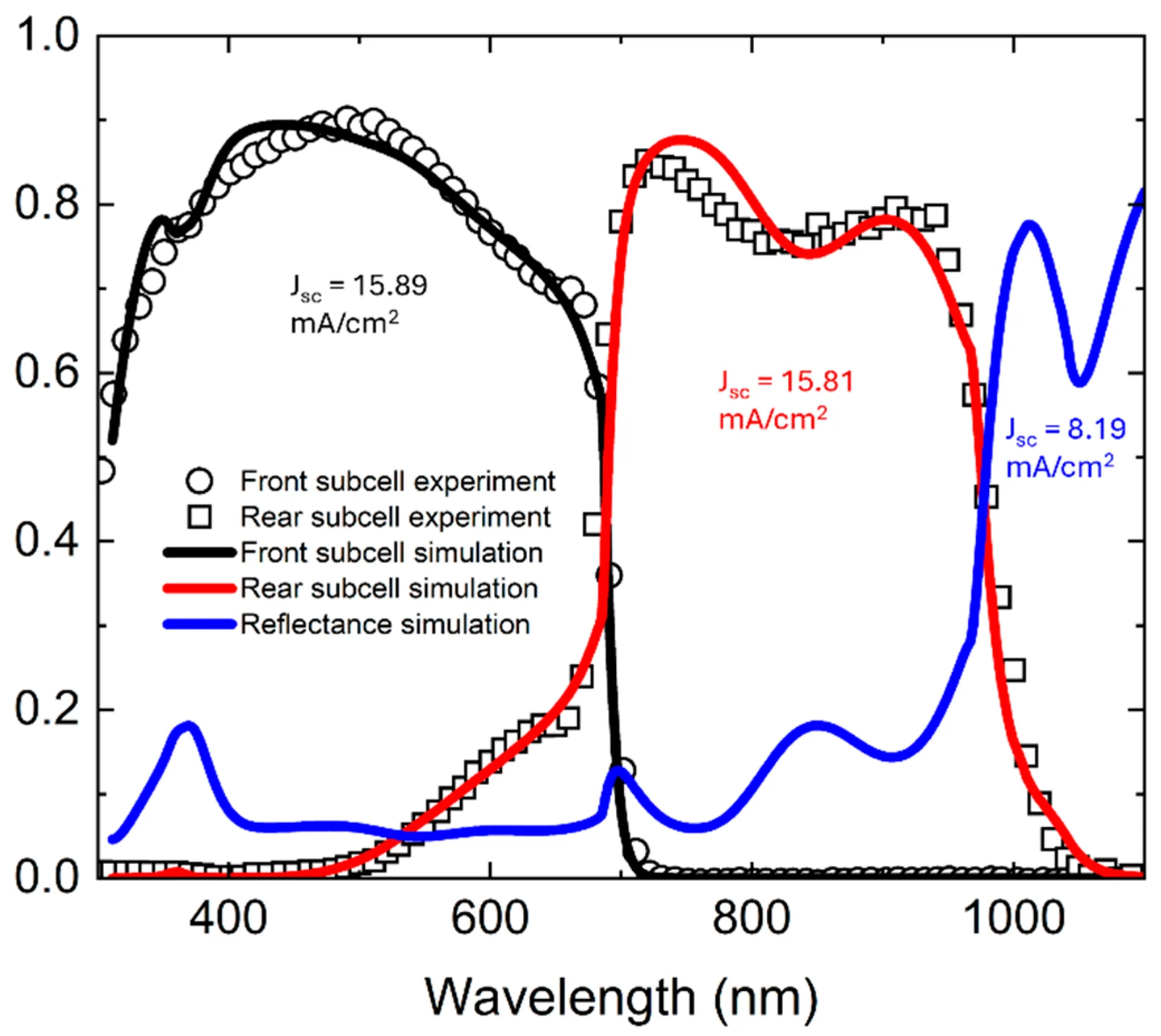
Simulated EQE (black and red lines) and reflectance (blue line) spectra of an all-perovskite tandem superstrate PV device compared with experimental spectra (symbols) measured by Wen et al. Short circuit current density (J_sc_) of each subcell and J_sc_ loss due to reflectance calculated under AM 1.5 illumination from the simulation are also shown. J_sc_ for the top and bottom subcells of 15.89 and 15.81 mA/cm^2^ from simulation are comparable to the experimentally measured J_sc_ of 15.9 and 16.0 mA/cm^2^ reported by Wen et al. [3].

The structural model of the all-perovskite device, shown in Figure 1, consists of 1 mm glass superstrate/115 nm indium-doped tin oxide (ITO)/10 nm effective medium approximation consisting of equal parts ITO and NiO_x_ with a monolayer of Me-4PACz/350 nm wide E_g_ perovskite where the E_g_ is 1.8 eV and the Urbach energy is 15 meV/20 nm fullerine (C_60_)/20 nm of SnO_2_ deposited by atomic layer deposition/2 nm Au/30 nm PEDOT:PSS/975 nm narrow E_g_ perovskite where the Eg is 1.29 eV and the Urbach energy is 23 meV/20 nm C_60_/7 nm bathocuproine/100 nm Cu. The E_g_ of the wide E_g_ perovskite is chosen to match what is reported in Wen et al.; the narrow E_g_ and Urbach energies are chosen to reproduce the shape of the experimental spectrum [3]. The front 25 nm of wide E_g_ perovskite nearest to the hole transport layer (HTL) is assumed to have a reduced carrier collection probability of 90% to better approximate the experimental spectrum [5,6,7]. These simulated spectra compared with the experimental spectra from Wen et al. are shown in Figure 2 [3]. Layer thicknesses are determined by initially using nominal thicknesses as reported in Wen et al., then adjusting as needed to better approximate the experimental spectrum. Wen et al. report the Au layer as 1 nm thick, but 2 nm Au thicknesses produce better agreement. Nominal thicknesses for the ITO, NiO_x_, perovskites, and PEDOT:PSS are not reported by Wen et al., so typical thicknesses from the previous literature are chosen and amended to produce agreement with the experimental EQE spectrum [3,8]. Altering these layers’ thicknesses by 5 nm or more produces a noticeable change in the simulated spectrum. A variable thickness MgF_2_ anti-reflection coating is applied on top of the 1 mm glass superstrate in additional simulations.

**Figure 3:**
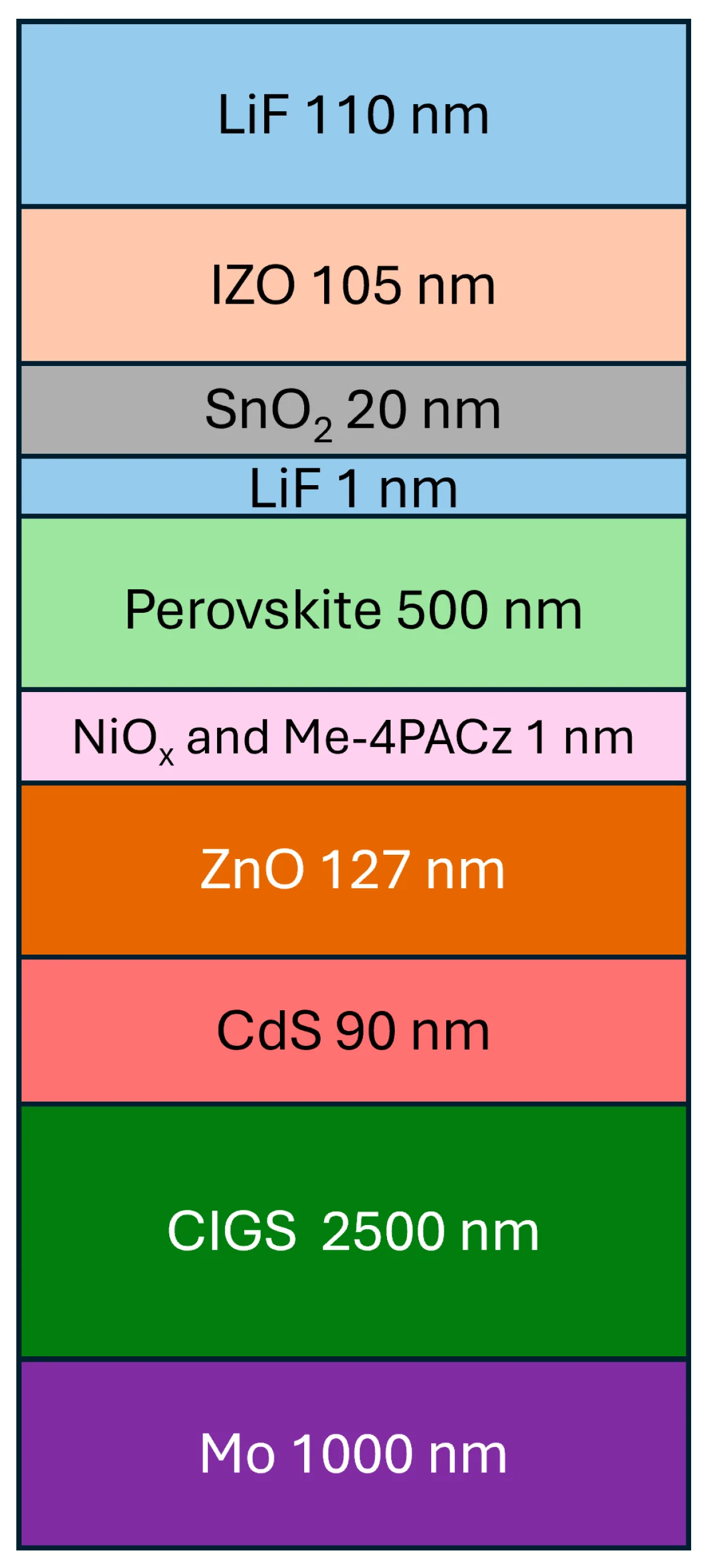
Nominal structural model used to simulate EQE spectra of a perovskite/copper indium gallium diselenide (CIGS) tandem substrate PV device. Thicknesses are chosen to approximate the EQE published by Jošt et al. [4].

**Figure 4:**
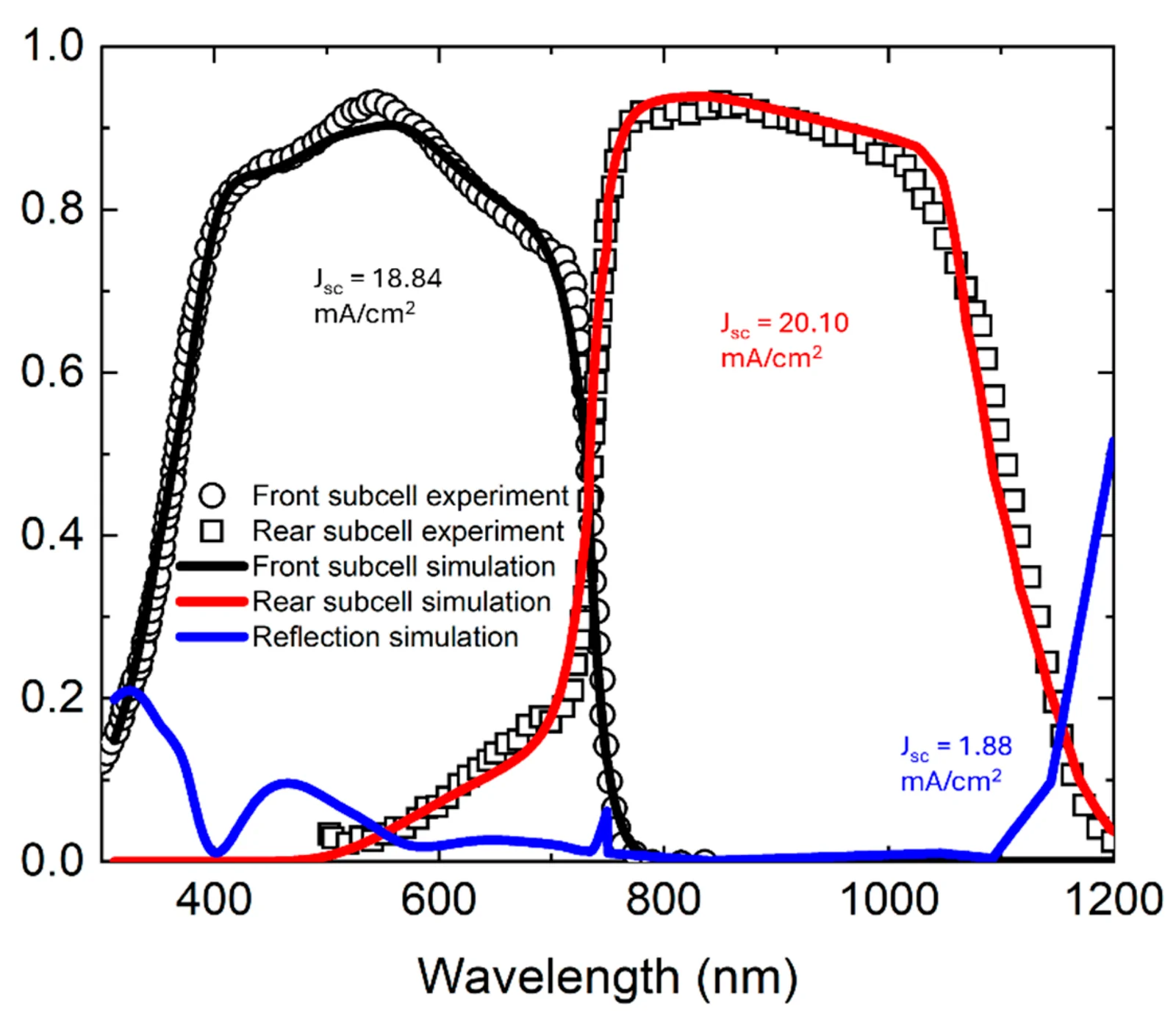
Simulated EQE (black and red lines) and reflectance (blue line) spectra of a perovskite/CIGS tandem superstrate PV device compared with experimental spectra (symbols) measured by Jost et al. J_sc_ of each subcell and J_sc_ loss due to reflectance calculated under AM 1.5 illumination from the simulation are also shown. J_sc_ for the top and bottom subcells of 18.84 and 20.10 mA/cm^2^ from simulation are comparable to the experimentally measured J_sc_ of 19.0 and 20.0 mA/cm^2^ reported by Jošt et al. [4].

Using results from our previous analyses of perovskite and CIGS solar cells, an initial model is created to approximate the experimental EQE spectra presented by Jošt et al., including optical properties of the perovskite and CIGS absorber layers [3,4,12]. The structural model of the perovskite/CIGS device is shown in Figure 3 and consists of 1 μm molybdenum (Mo) on a soda lime glass substrate/2500 nm CIGS/90 nm cadmium sulfide (CdS)/127 nm zinc oxide (ZnO)/1 nm nickel oxide (NiO_x_) with a monolayer of Me-4PACz/500 nm perovskite/1 nm lithium fluoride (LiF)/20 nm tin oxide (SnO_2_)/105 nm indium zinc oxide (IZO)/110 nm LiF. The glass substrate is not shown as the Mo layer is optically opaque over the near-infrared-to-ultraviolet spectral range of PV device operation. The perovskite layer has an E_g_ of 1.68 eV and an Urbach energy of 25 meV, and the CIGS layer has an E_g_ of 1.17 eV and an Urbach energy of 34.7 meV. Perovskite E_g_ is reported in Jost et al., and the Urbach energy is chosen to reproduce the shape of the experimental EQE spectrum. CIGS E_g_ and Urbach energy are calculated from Aryal et al. assuming x = 0.2 to match the experimental EQE spectrum [12]. The rear 50 nm of perovskite adjacent to the NiO_x_ with Me-4PACz and rear 1000 nm of CIGS adjacent to the Mo are assumed to have reduced carrier collection probabilities of 0%, and the bulk CIGS has reduced carrier collection probability of 90% to better approximate the experimental EQE spectrum. These regions of reduced carrier collection probability coincide with the nucleation layers that occur during thin film growth where more disordered, smaller grained, lower electronic quality material is expected [5,6,7]. Although the modeling approach cannot distinguish explicitly between bulk recombination, interface recombination, and back-contact losses, it does provide the physical locations where electronic losses occur. These simulated spectra compared with the experimental spectra from Jošt et al. are shown in Figure 4 [4].

In additional simulations that vary the absorber layer thicknesses, it is assumed that the regions of reduced carrier collection probabilities maintain the same thicknesses for both tandem device structures. This is a reasonable assumption, as changes in the bulk perovskite thickness are not expected to significantly change the nucleation layer thickness [7].

Optical properties of all simulated materials are obtained from the literature. Perovskite optical properties come from Amonette et al. with revised E_g_ and Urbach energies, and NiO_x_ plus the Me-4PACz optical properties are also from Amonette et al. [8]. ITO, PEDOT:PSS, BCP and C_60_ optical properties are published in Subedi et al., and SnO_2_ optical properties are from Bordovalos et al. [5,6]. Au and Cu optical properties are obtained from the Handbook of Optical Properties of Solids [13]. CIGS optical properties are calculated as described in Aryal et al. assuming x = 0.2 [12]. ZnO optical properties are published in Uprety et al., and CdS optical properties are obtained from Sapkota [14,15]. MgF_2_ and Mo optical properties come from Hara et al., and LiF optical properties are from Li et al. (1976) [16,17]. CdS optical properties are from Li et al. (2010) [18]. IZO optical properties are obtained from spectroscopic ellipsometry analysis, which is discussed in the Supplementary Materials.

## 3. Results and Discussion

J_sc_ is calculated by integrating the product of the EQE spectra with the solar irradiance spectrum with respect to wavelength across the spectral range where substantial absorption occurs in the semiconductor absorber layer. For the all-perovskite tandem device, the calculated J_sc_ values from simulated EQE spectra under the AM 1.5 solar spectrum are 15.89 mA/cm^2^ for the wide E_g_ subcell and 15.81 mA/cm^2^ for the narrow E_g_ subcell, which are comparable to Wen et al.’s reported values of 15.9 and 16.0 mA/cm^2^, respectively [3]. Under the AM 0 irradiance, J_sc_ in the wide and narrow E_g_ perovskite subcells are predicted to be 20.21 and 18.65 mA/cm^2^, respectively. For the CIGS/perovskite tandem device, calculated J_sc_ from simulated EQE spectra under the AM 1.5 solar spectrum are 18.86 mA/cm^2^ for the perovskite subcell and 20.37 mA/cm^2^ for the CIGS subcell, which are comparable to Jošt et al.’s reported values of 19.0 and 20.0 mA/cm^2^ [4]. Under the AM 0 illumination, J_sc_ is predicted to be 23.22 mA/cm^2^ in the perovskite subcell and 23.41 mA/cm^2^ in the CIGS subcell.

**Figure 5:**
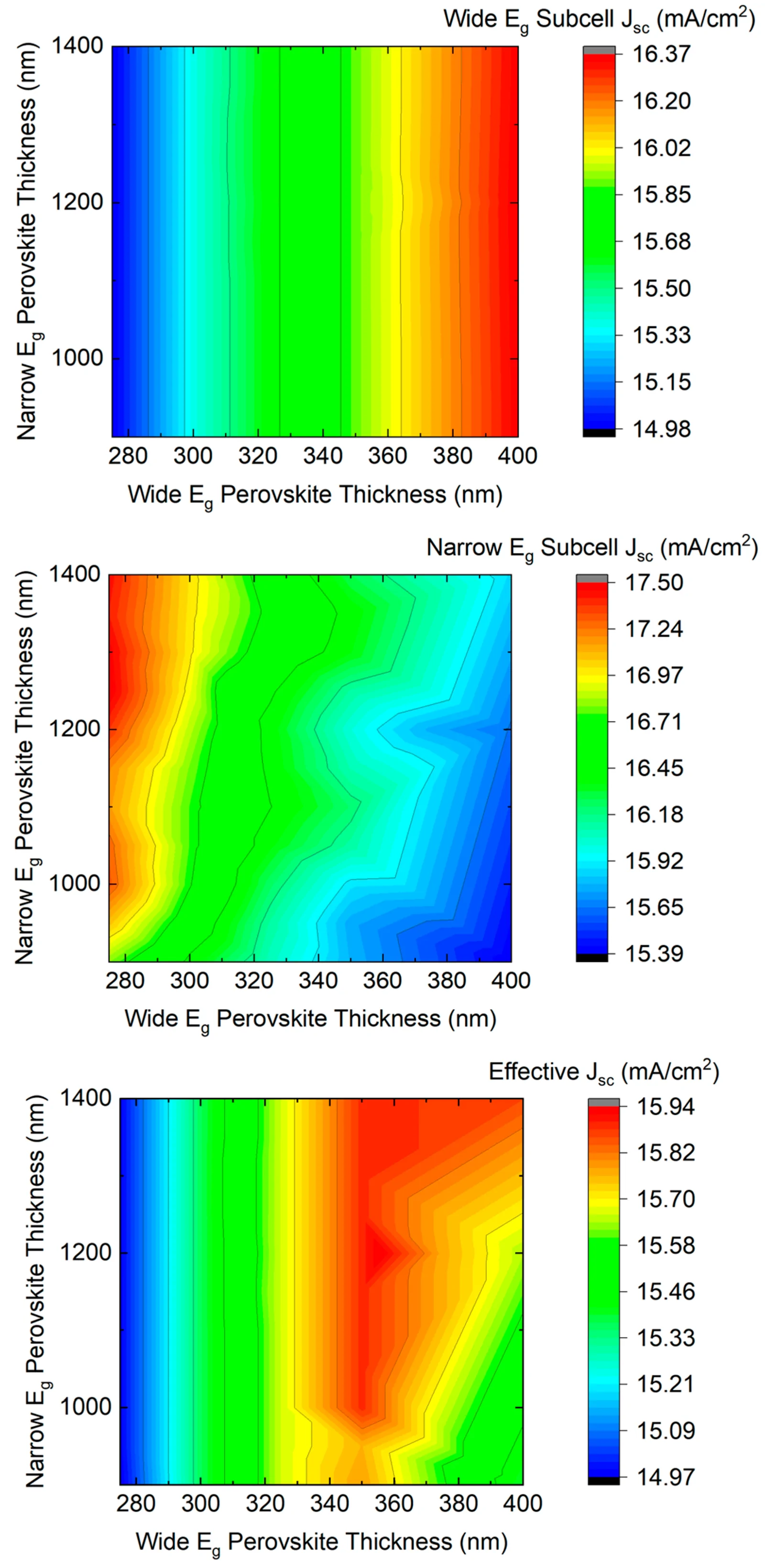
Calculated J_sc_ under AM 1.5 illumination of the wide E_g_ and narrow E_g_ subcells of an all-perovskite tandem device as well as the effective J_sc_ of the tandem device as functions of absorber layer thicknesses.

**Figure 6:**
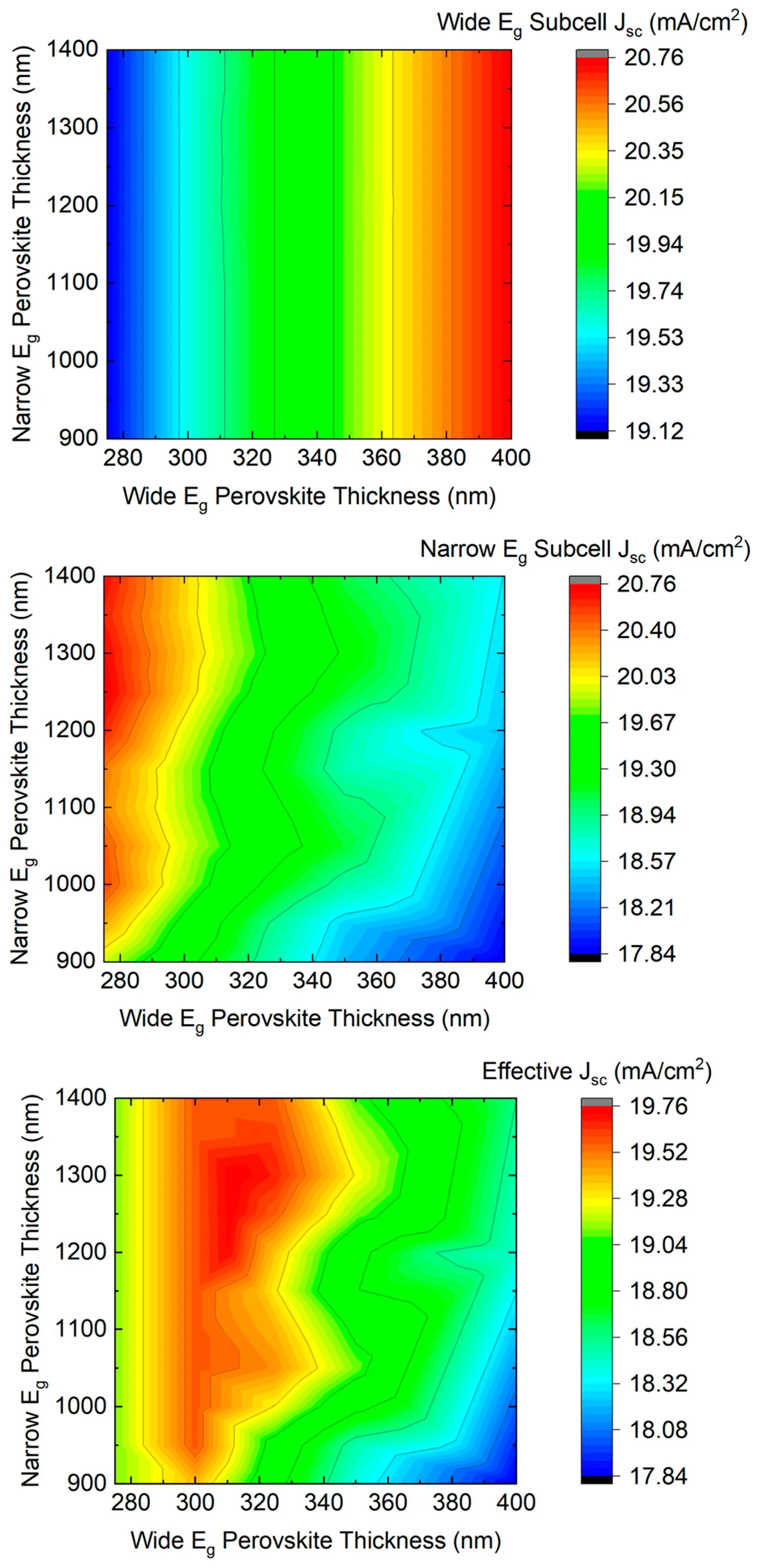
Calculated J_sc_ under AM 0 illumination of the wide E_g_ and narrow E_g_ subcells of an all-perovskite tandem device as well as the effective J_sc_ of the tandem device as functions of absorber layer thicknesses.

**Figure 7:**
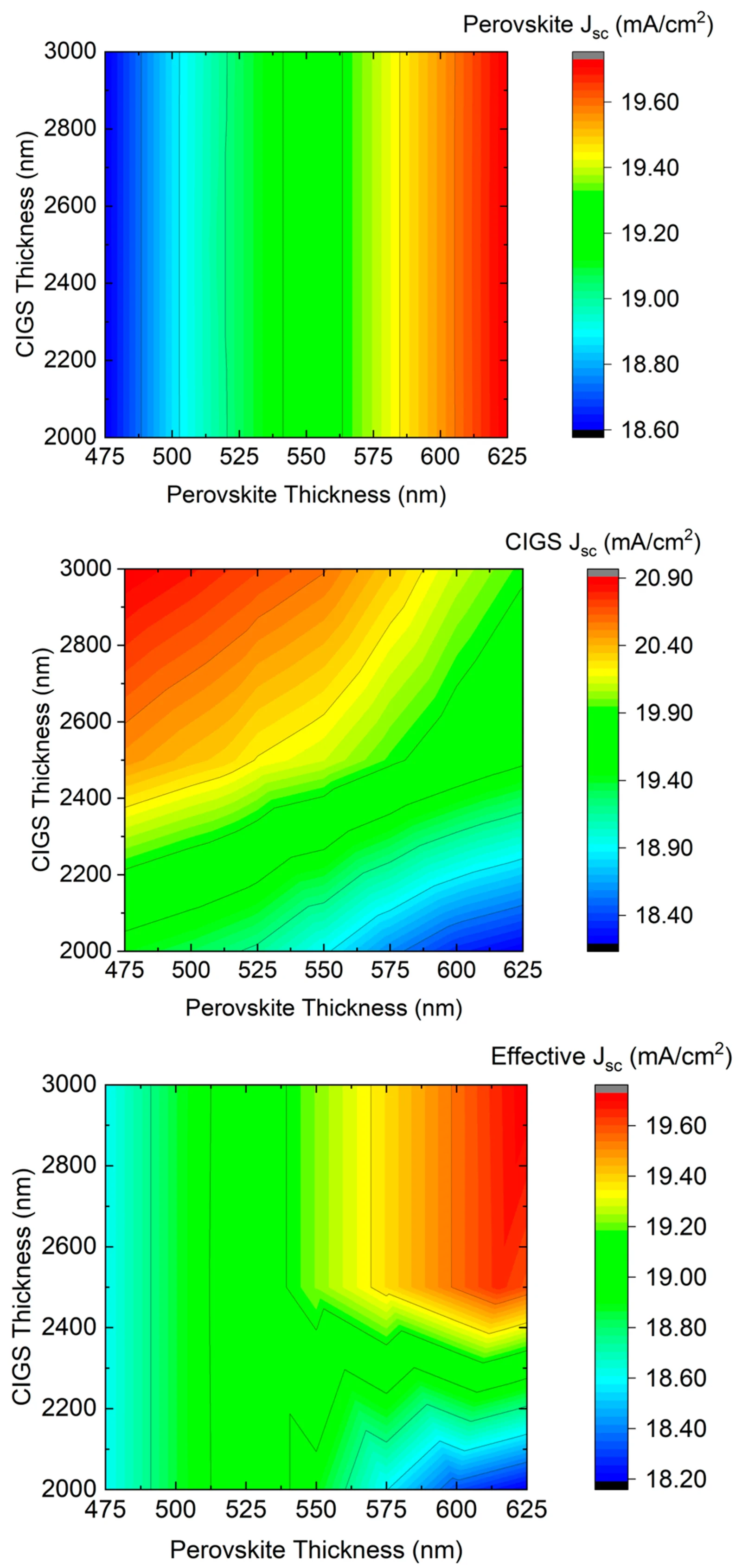
Calculated J_sc_ under AM 1.5 illumination of the perovskite and CIGS subcells of a tandem device as well as the effective J_sc_ of the tandem device as functions of absorber layer thicknesses.

**Figure 8:**
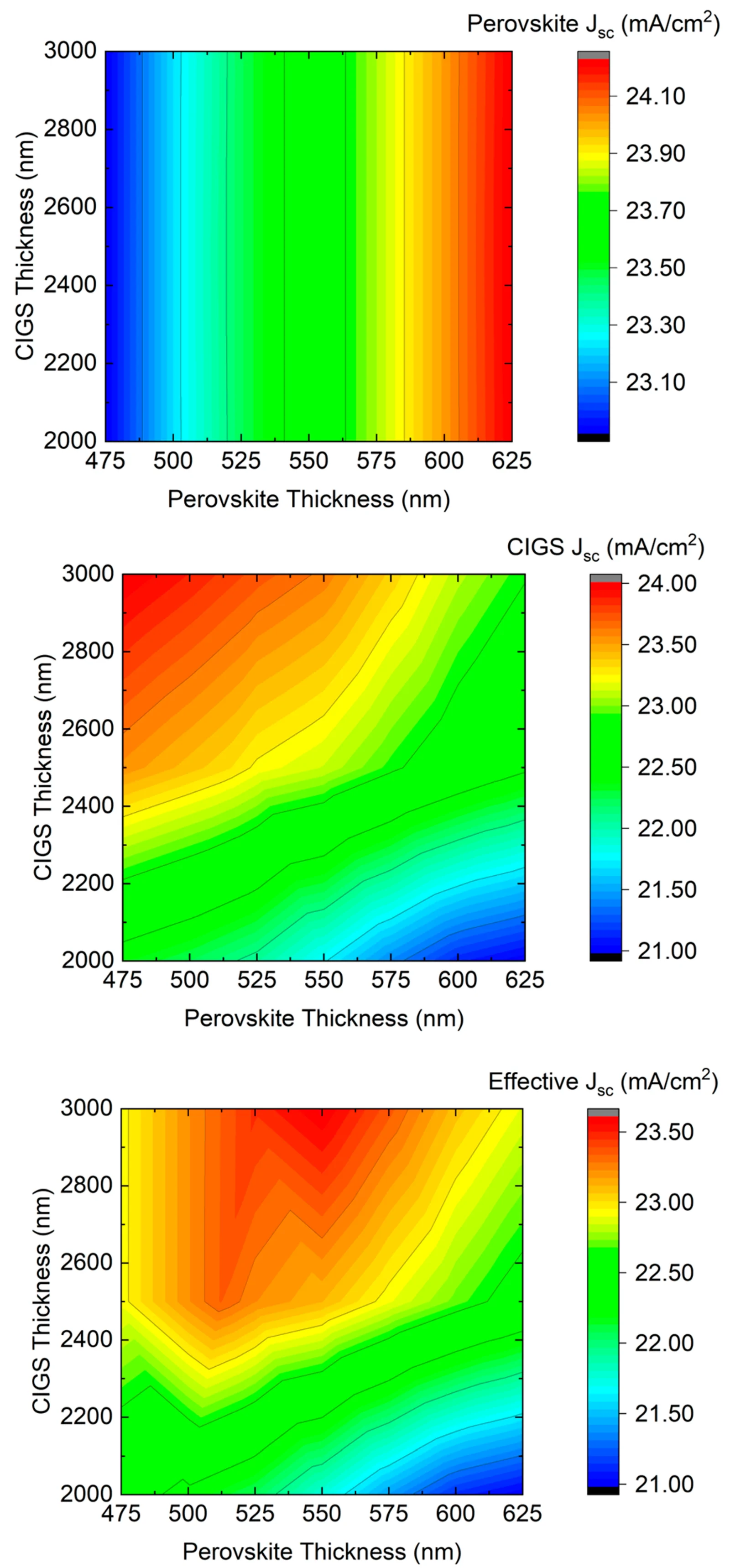
Calculated J_sc_ under AM 0 illumination of the perovskite and CIGS subcells of a tandem device as well as the effective J_sc_ of the tandem device as functions of absorber layer thicknesses.

Simulations are repeated with combinations of absorber layer thicknesses with all other parameters fixed to identify practical current matching thicknesses under both AM 1.5 and AM 0 solar irradiance. The resulting J_sc_ for the all-perovskite device under AM 1.5 irradiance are shown in Figure 5, the all-perovskite device under AM 0 is shown in Figure 6, the perovskite/CIGS device under AM 1.5 is shown in Figure 7, and the perovskite/CIGS device under AM 0 is shown in Figure 8.

For the all-perovskite device, narrow E_g_ perovskite layer thicknesses of up to 1400 nm are simulated, but 1200 nm is chosen as a realistic practical upper limit based on the previous literature [19,20,21]. Tandem device J_sc_ is limited by the lowest subcell J_sc_. Under AM 1.5 illumination, a wide E_g_ perovskite thickness of 356 nm and narrow E_g_ perovskite thickness of 1200 nm yields the highest device J_sc_ of 15.94 mA/cm^2^, and under AM 0 illumination, a wide E_g_ perovskite thickness of 310 nm and narrow E_g_ perovskite thickness of 1200 nm yields the highest device J_sc_ of 19.72 mA/cm^2^. Assuming the same V_oc_ and FF as reported in Wen et al., these J_sc_ values correspond to device efficiencies of 26.05% for the AM 1.5 illumination and 32.22% for the AM 0 illumination. These values are an improvement over the efficiencies calculated using nominal absorber layer thicknesses, which are 25.83% for the AM 1.5 illumination and 30.33% for the AM 0 illumination. The experimentally measured efficiency reported by Wen et al. under AM 1.5 illumination is 26.0%. This is close to the calculated value of 25.83%, and the discrepancy can be attributed to the use of material optical properties already available in the literature rather than the exact optical properties found in the specific device and the structural model applied to describe the device. Regardless of these shortcomings, J_sc_ are sufficiently close and enable the use of this model to describe the EQE of a high-performance all-perovskite tandem solar cell.

For the perovskite/CIGS device, CIGS layer thicknesses of up to 3000 nm are simulated, but 2500 nm is chosen as a realistic upper limit to CIGS thickness, as Jošt et al. uses a 2500 nm CIGS thickness and CIGS thicknesses exceeding 2500 nm in the literature are uncommon [4]. Under AM 1.5 illumination, a perovskite thickness of 615 nm and CIGS thickness of 2500 nm yields the highest device J_sc_ of 19.65 mA/cm^2^, and under AM 0 illumination, a perovskite thickness of 512 nm and CIGS thickness of 2500 nm yields the highest device J_sc_ of 23.34 mA/cm^2^. Assuming the same Voc and FF as reported in Jošt et al., these J_sc_ values correspond to device efficiencies of 24.76% for the AM 1.5 case and 29.41% for the AM 0 case. These values are an improvement over the efficiencies calculated using nominal absorber layer thicknesses, which are 23.74% for the AM 1.5 illumination and 29.15% for the AM 0 illumination. The calculated value under AM 1.5 illumination of 23.74% is comparable to Jošt et al.’s experimentally measured value of 24.2%, and the discrepancy can again be attributed to the use of material optical properties already available in the literature rather than the exact optical properties found in the specific device and the structural model applied to describe the device.

The effects of anti-reflective coating inclusion and thickness for both devices are simulated while absorber thicknesses are kept at their ideal values discussed previously. For the all-perovskite device, optical properties of thin film MgF_2_ from Hara et al. are used to simulate the anti-reflective coating [16]. The perovskite/CIGS device is simulated with LiF anti-reflective coating in accordance with Jošt et al. [4].

**Figure 9:**
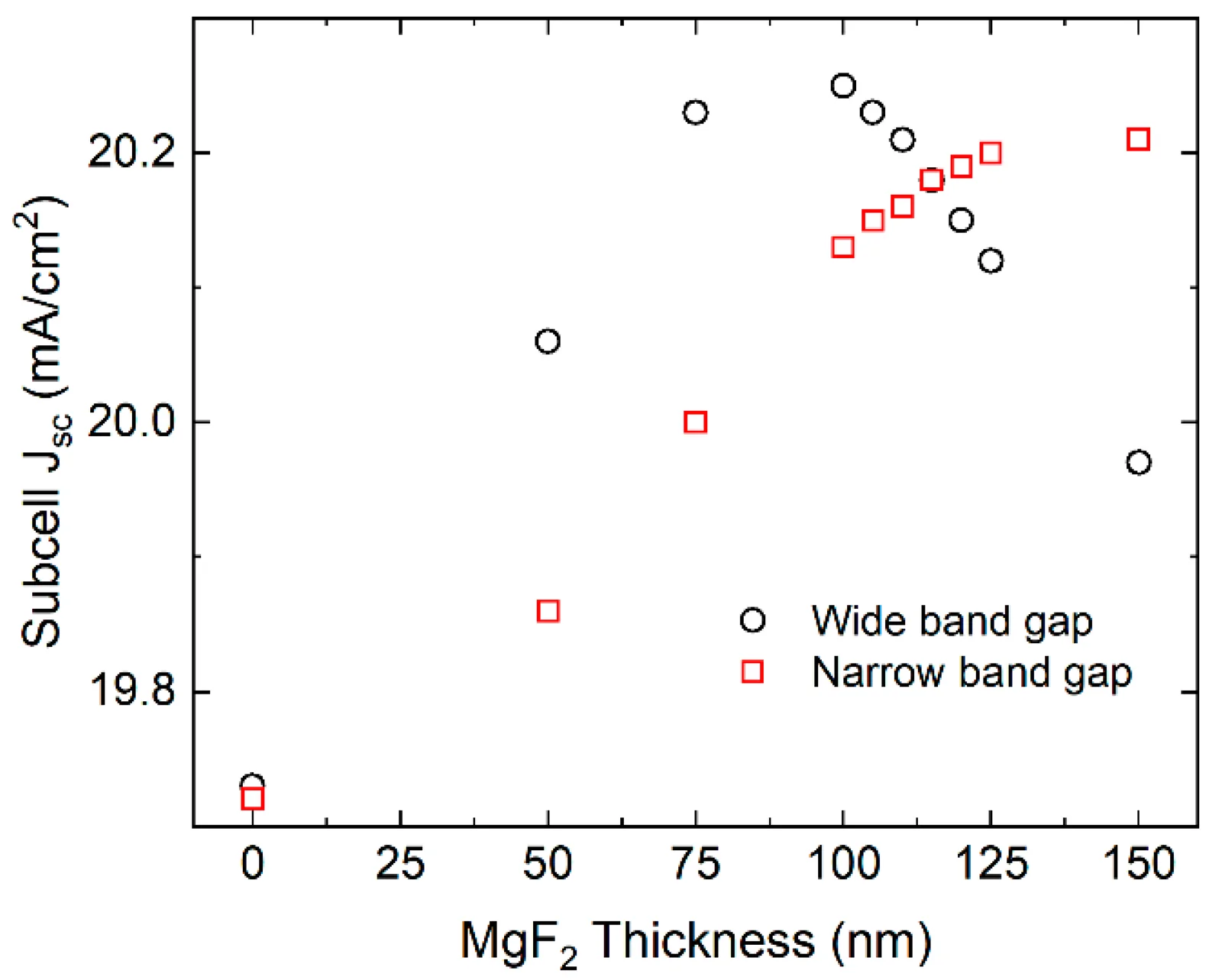
Calculated J_sc_ as a function of MgF_2_ anti-reflection coating film thickness of wide and narrow E_g_ subcells of an all-perovskite tandem PV device where the wide E_g_ perovskite is 310 nm thick and the narrow E_g_ perovskite is 1200 nm thick under AM 0 illumination. The narrow E_g_ thickness is chosen as this is the thickest perovskite absorber layer in a tandem device reported in the literature, and the wide E_g_ thickness is chosen to produce current matching conditions under AM 0 illumination.

**Figure 10:**
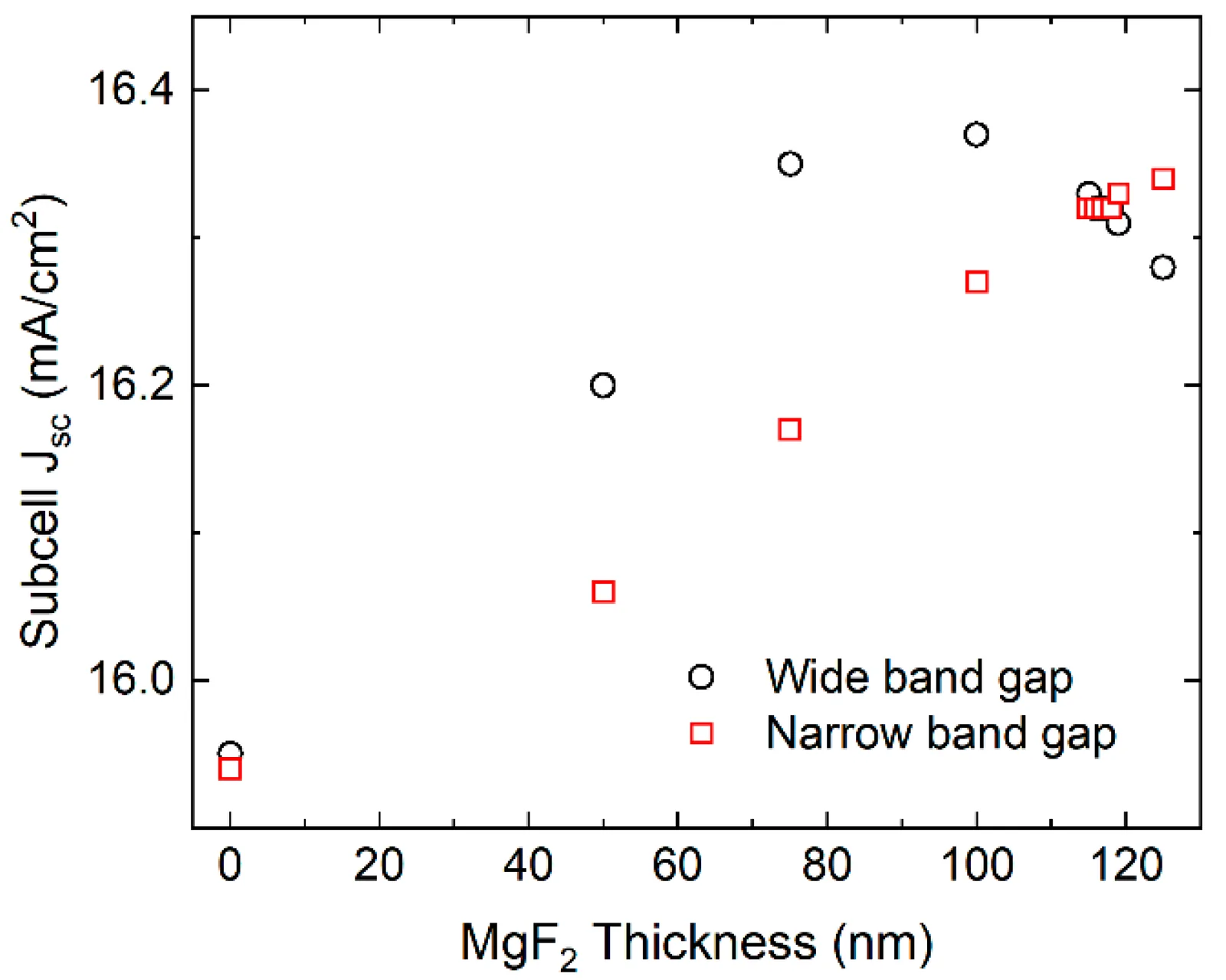
Calculated J_sc_ as a function of MgF_2_ anti-reflection coating film thickness of wide and narrow E_g_ subcells of an all-perovskite tandem PV device where the wide E_g_ perovskite is 356 nm thick and the narrow E_g_ perovskite is 1200 nm thick under AM 1.5 illumination. The narrow E_g_ thickness is chosen as this is the thickest perovskite absorber layer in a tandem device reported in the literature, and the wide E_g_ thickness is chosen to produce current matching conditions under AM 1.5 illumination.

For the all-perovskite device under AM 0 illumination, adding a 115 nm thick MgF_2_ coating increases the J_sc_ to 20.18 mA/cm^2^ as shown in Figure 9 and Table 1, which corresponds to an efficiency of 32.84%, slightly higher than 32.22% without the anti-reflective coating. Under AM 1.5 illumination, a 115–118 nm thick MgF_2_ anti-reflective coating on top of the glass superstrate increases the J_sc_ to 16.32 mA/cm^2^ as shown in Figure 10 and Table 2 which corresponds to an efficiency of 26.67%, again slightly higher than 26.05% without anti-reflective coating. Although these increases in J_sc_ are greater than the error between the experimental and simulated values, this small increase in J_sc_ is likely not substantial. It is also prudent to consider that small variations in performance parameters can exist across nominally identical devices [9]. However, these calculations reveal the trend that occurs with increasing MgF_2_ thickness in this device structure.

**Figure 11:**
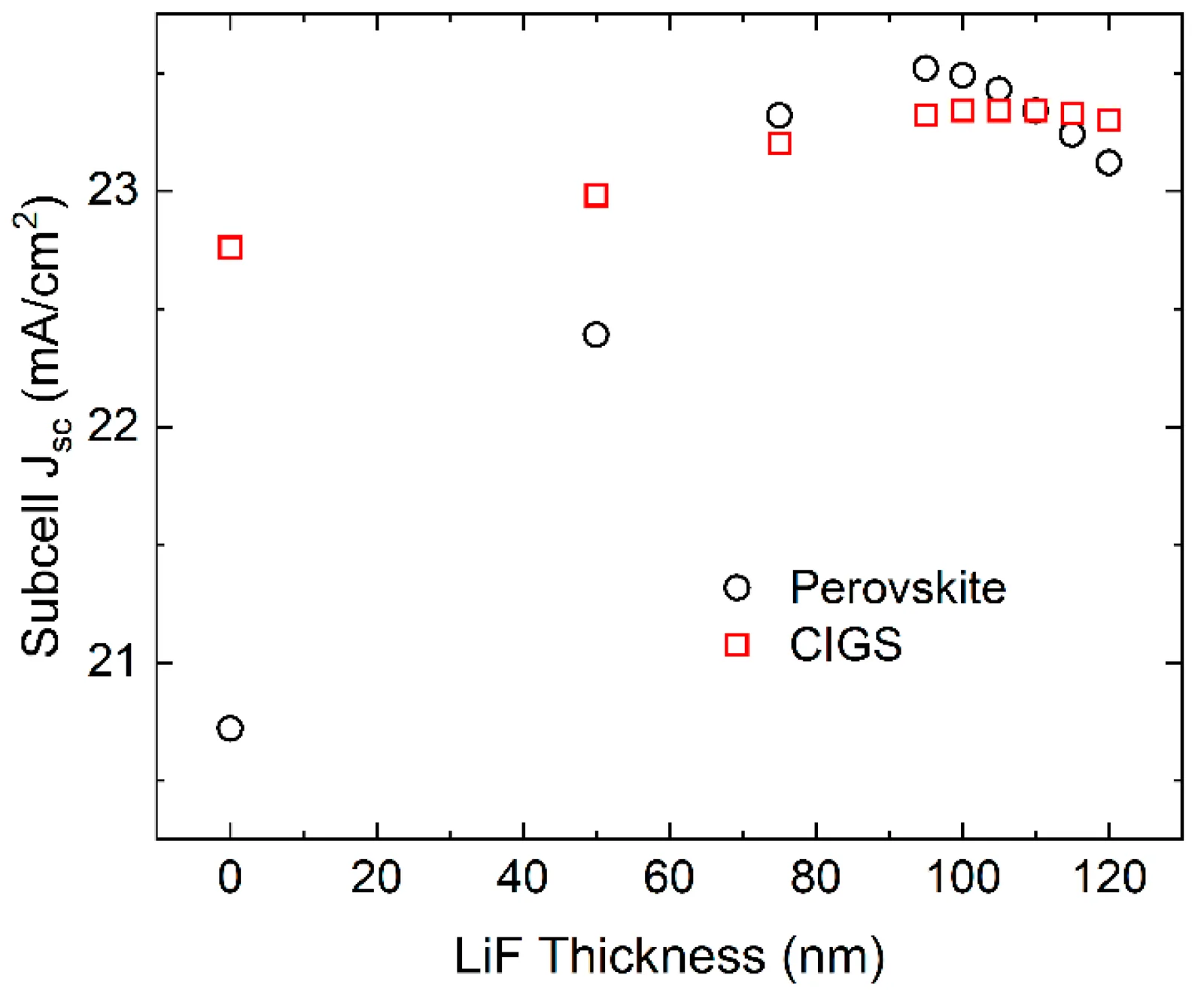
Calculated J_sc_ as a function of LiF anti-reflection coating film thickness of perovskite and CIGS subcells in a tandem PV device where the perovskite is 512 nm thick and the CIGS layer is 2500 nm thick under AM 0 illumination. The CIGS thickness is chosen to match the literature, and the perovskite thickness is chosen to produce current matching conditions under AM 0 illumination.

**Figure 12:**
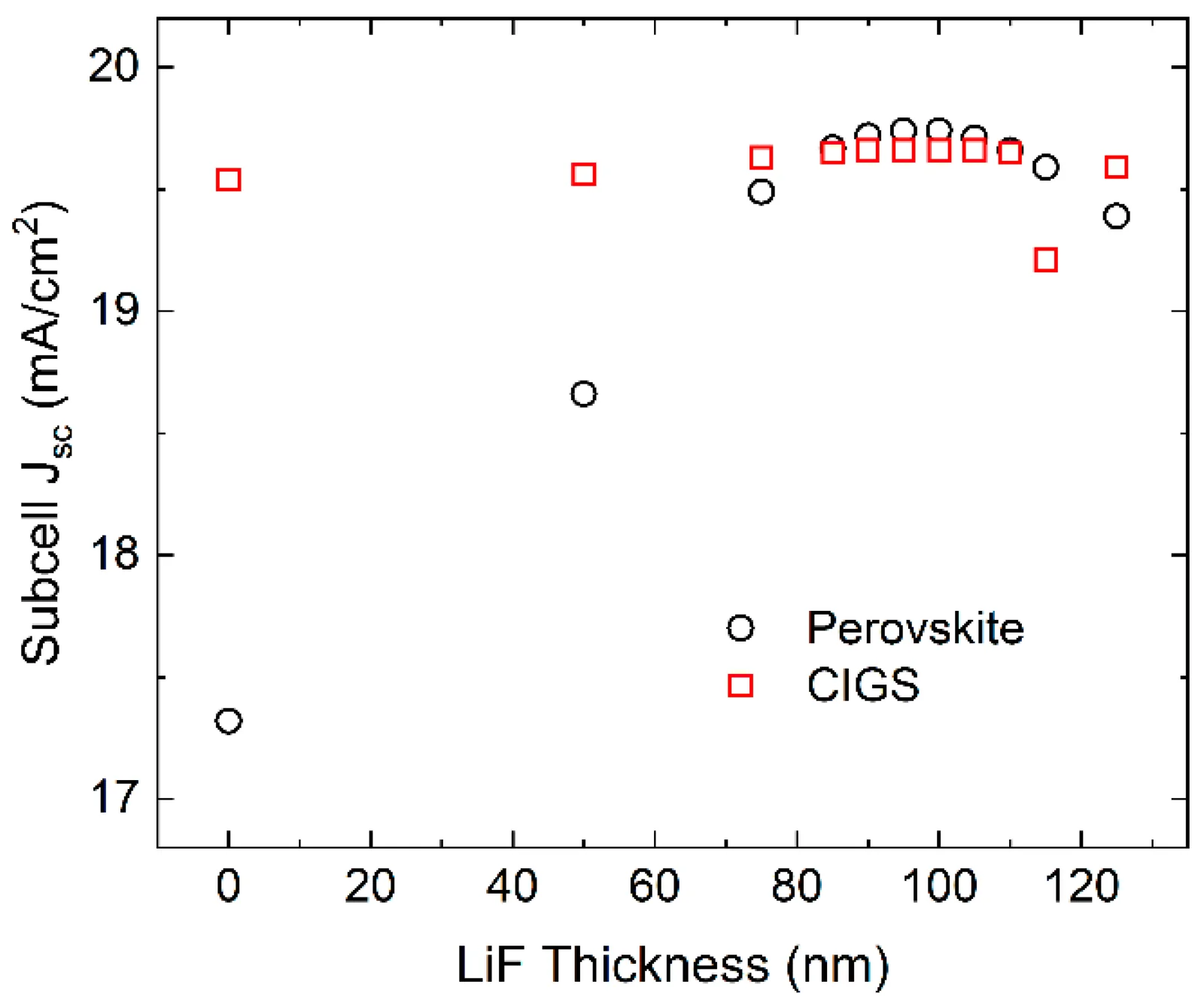
Calculated J_sc_ as a function of LiF anti-reflection coating film thickness of perovskite and CIGS subcells in a tandem PV device where the perovskite is 615 nm thick and the CIGS layer is 2500 nm thick under AM 1.5 illumination. The CIGS thickness is chosen to match the literature, and the perovskite thickness is chosen to produce current matching conditions under AM 1.5 illumination.

The perovskite/CIGS device is simulated with a 110 nm thick LiF anti-reflective coating as in Jošt et al. [4]. Under AM 0 illumination, this is the ideal LiF thickness to use with the absorber layer thicknesses here as shown in Figure 11 and Table 3, although any LiF thickness between 100 and 110 nm yields the same effective J_sc_. Under AM 1.5 illumination, although approximate current matching occurs at 110 nm LiF, a slightly higher effective J_sc_ can be achieved when the LiF thickness is between 90 and 105 nm as shown in Figure 12 and Table 4. Assuming the same values for V_oc_ and FF as reported in Jošt et al., the efficiency at 110 nm LiF would be 24.76% while LiF thickness between 90 and 105 nm would yield 24.78% efficiency.

**Figure 13:**
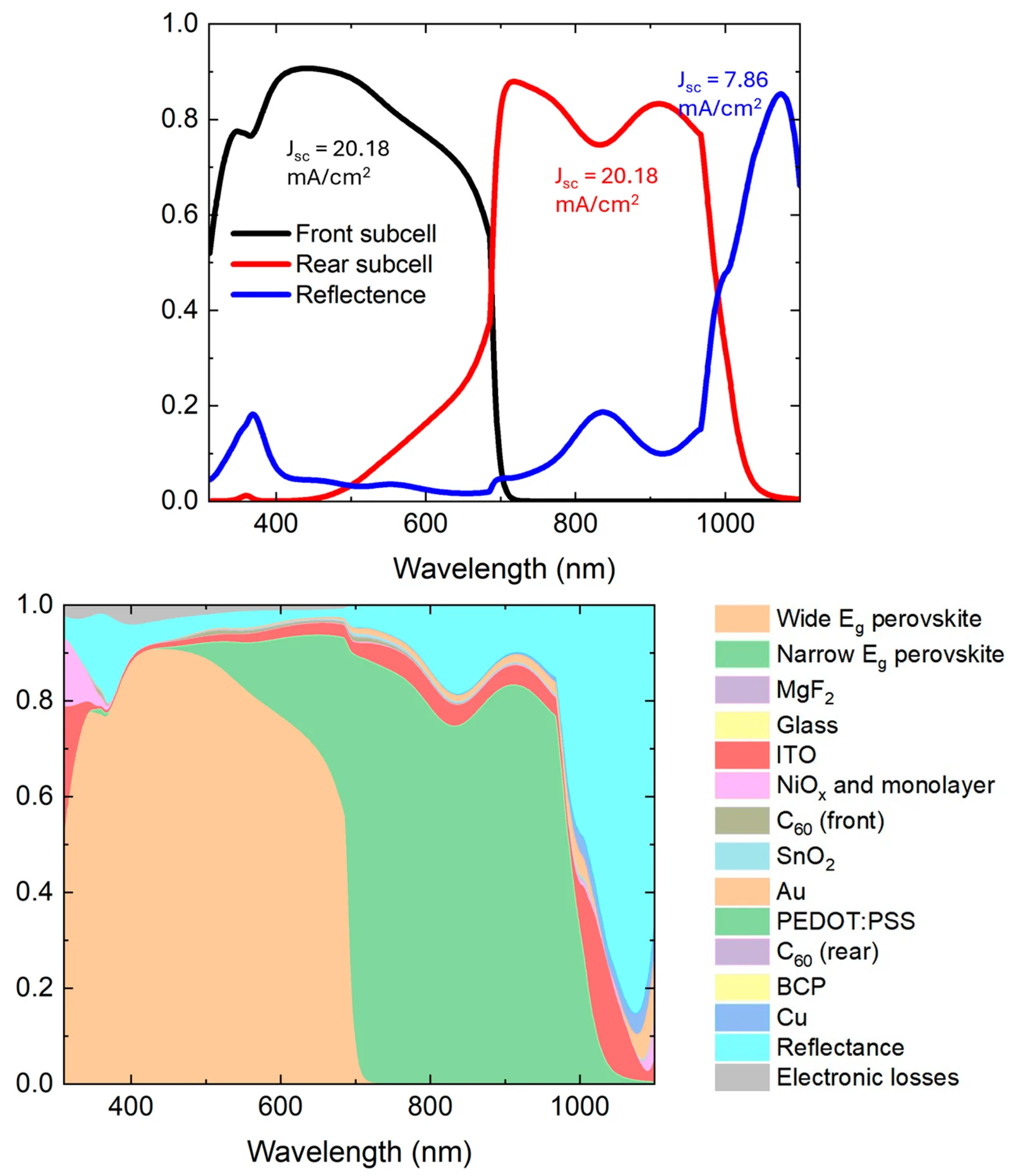
Simulated EQE and reflection spectra of the optimized all-perovskite tandem PV device under AM 0 illumination (**top**) and absorbance of each layer of the device as well as reflectance and electronic losses (**bottom**). The optimized device is simulated as having 115 nm MgF_2_ anti-reflective coating, 310 nm thick wide E_g_ perovskite, and 1200 nm thick narrow E_g_ perovskite.

**Figure 14:**
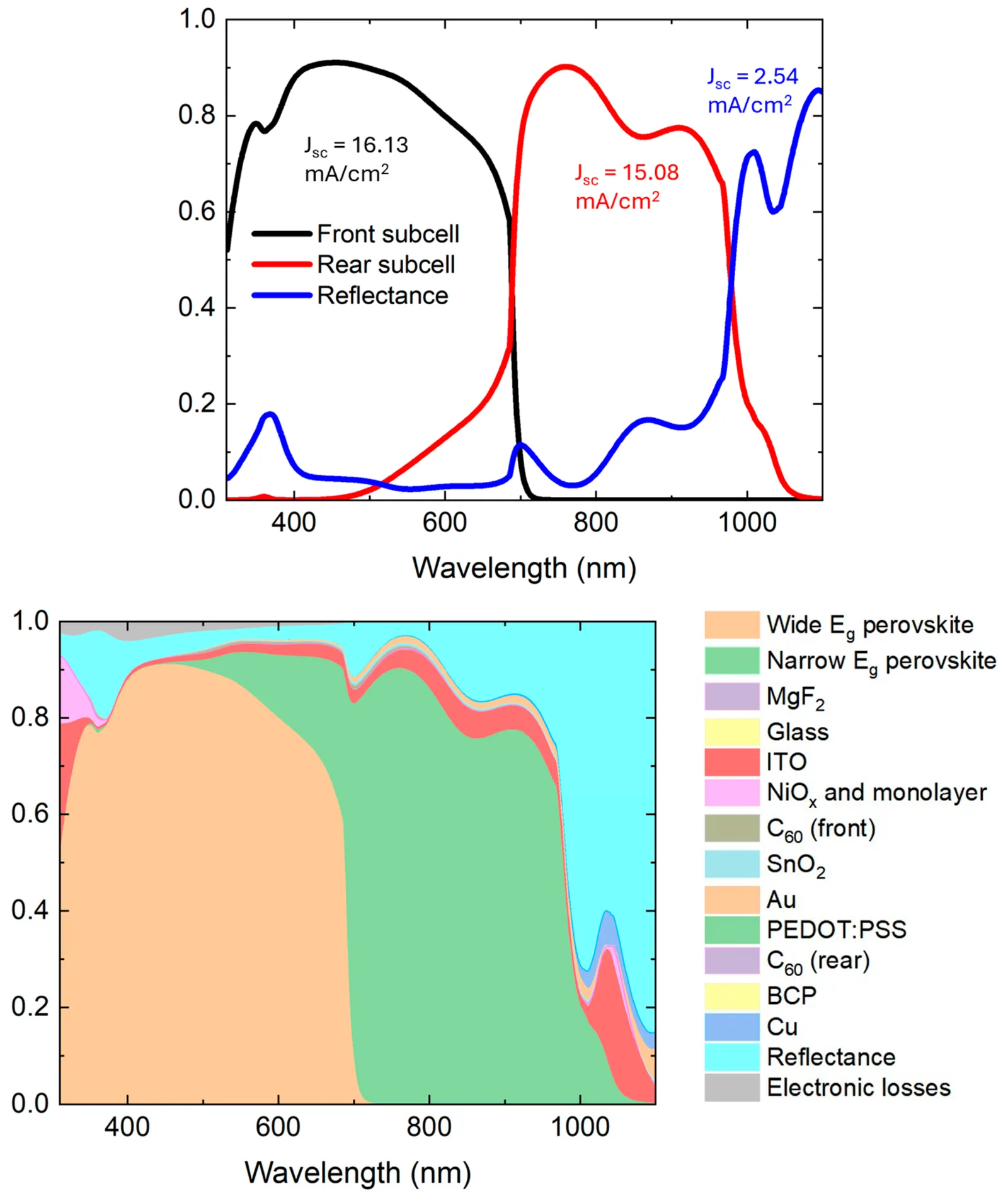
Simulated EQE and reflection spectra of the optimized all-perovskite tandem PV device under AM 1.5 illumination (**top**) and absorbance of each layer of the device as well as reflectance and electronic losses (**bottom**). The optimized device is simulated as having 117 nm MgF_2_ anti-reflective coating, 356 nm thick wide E_g_ perovskite, and 1200 nm thick narrow E_g_ perovskite.

**Figure 15:**
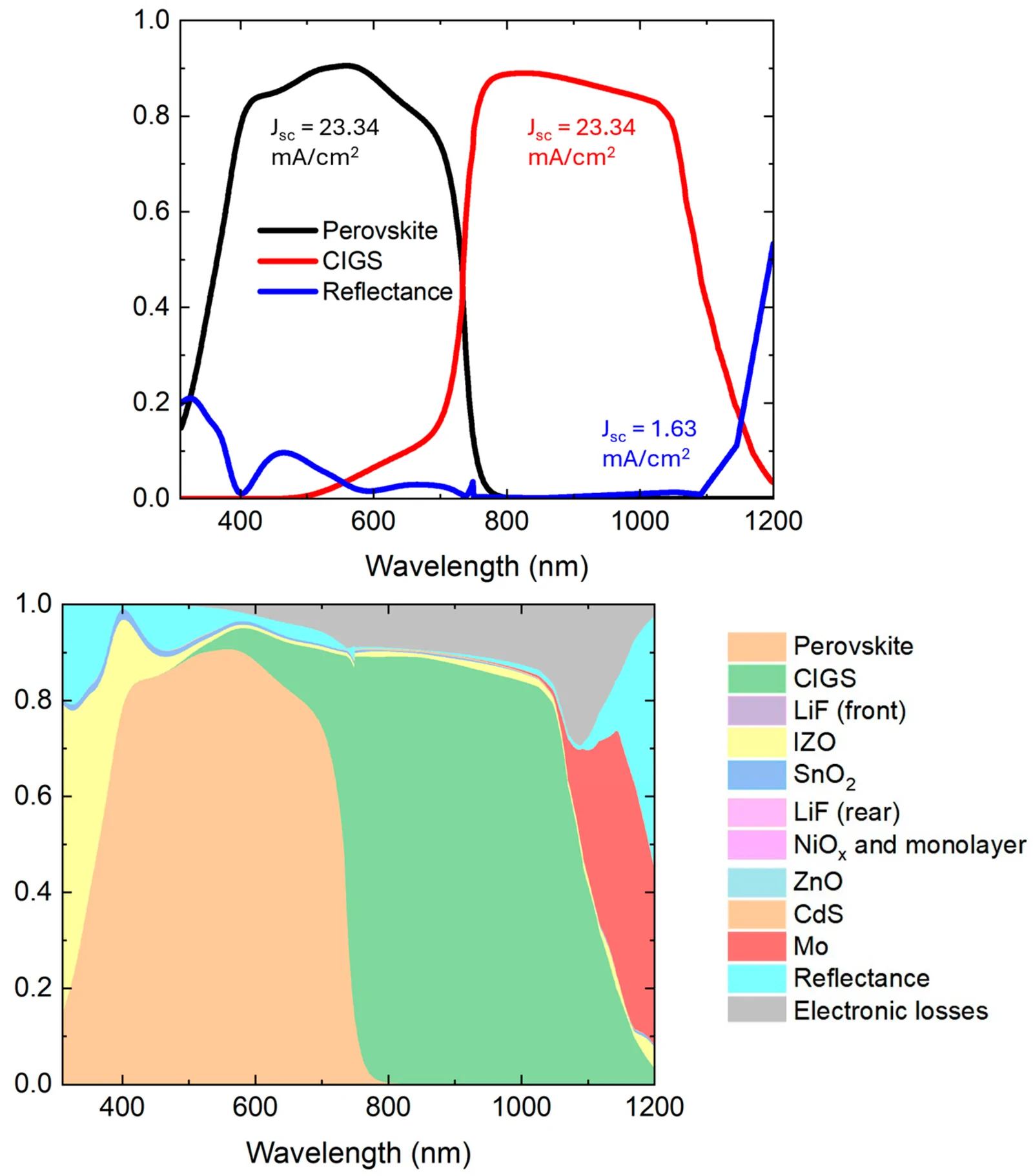
Simulated EQE and reflection spectra of the optimized CIGS/perovskite tandem PV device under AM 0 illumination (**top**) and absorbance of each layer of the device as well as reflectance and electronic losses (**bottom**). The optimized device is simulated as having 110 nm LiF anti-reflective coating, 512 nm thick perovskite, and 2500 nm thick CIGS.

**Figure 16:**
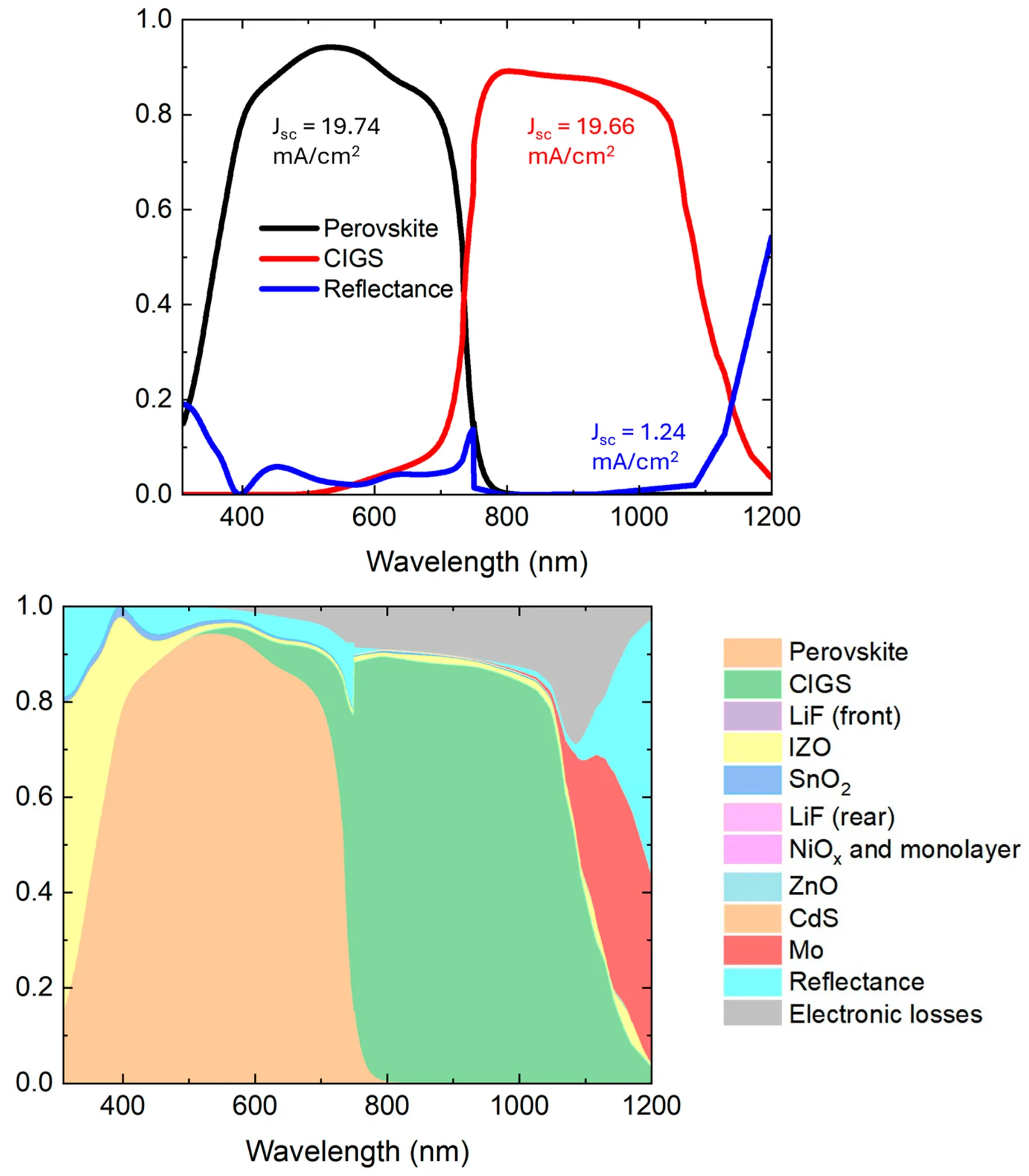
Simulated EQE and reflection spectra of the optimized CIGS/perovskite tandem PV device under AM 1.5 illumination (**top**) and absorbance of each layer of the device as well as reflectance and electronic losses (**bottom**). The optimized device is simulated as having 95 nm LiF anti-reflective coating, 615 nm thick perovskite, and 2500 nm thick CIGS.

Further simulations of each device using optimized absorber layer and anti-reflective coating layer thicknesses are performed and the EQE spectra, absorption spectra of each layer, reflectance, illuminance, and electronic losses are determined to serve as a basis for further J_sc_ optimization. Results for the all-perovskite device under AM 0 illumination are shown in Figure 13 and Table 5, results for the all-perovskite device under AM 1.5 illumination are shown in Figure 14 and Table 6, results for the CIGS/perovskite device under AM 0 are shown in Figure 15 and Table 7, and results for the CIGS/perovskite device under AM 1.5 are shown in Figure 16 and Table 8. These results serve as a starting point for further optimization. For example, in the all-perovskite devices, there is substantial reflection loss equivalent to 9.18 mA/cm^2^ of lost current for the device under AM 1.5 illumination and 7.86 mA/cm^2^ for the device under AM 0 illumination despite the simulated anti-reflective coating. Application of different anti-reflective materials, multilayer coatings, or texturing could improve device performance [22,23]. Additionally, the ITO exhibits the highest parasitic absorption of any layer in the device equivalent to a current loss of 2.48 mA/cm^2^ for the device under AM 1.5 illumination and 2.70 mA/cm^2^ for the device under AM 0 illumination; altering its thickness or replacing it with another transparent conducting oxide could also improve device performance [24,25]. The NiO_x_ and monolayer of Me4-PACz also exhibits substantial parasitic absorption at short wavelengths which limits the J_sc_ from the wide E_g_ perovskite top subcells, and either its modification or replacement could be a subject of future research [26]. The CIGS/perovskite device exhibits significant electronic losses of 4.17 mA/cm^2^ in the AM 0 case and 3.44 mA/cm^2^ in the AM 1.5 case which could be reduced by improving the quality of the CIGS layer [27,28,29]. The IZO layer also exhibits parasitic absorption at short wavelengths, which could be minimized by altering its thickness or replacing it with another transparent conducting oxide [24,25].

There are limitations to this methodology of using transfer matrix-based EQE simulations to model PV device J_sc_ and performance. These simulations do not directly calculate other key PV performance factors such as open circuit voltage and fill factor. Additionally, these simulations are purely based on optical calculations and do not account for parameters seen in real devices the way other simulation software such as SCAPS 1-D 3.3.10 might. In addition to complex dielectric function spectra, SCAPS 1-D can account for defect densities, carrier mobilities, resistances and more, though when calculating J_sc_, optical-based simulation software such as e-ARC produces results closer to experiment [8]. The optical properties used as input for each material in the simulations in this manuscript are obtained from available published spectra, so they may not perfectly represent the properties of the experimental device. The PCE calculations performed in this manuscript assume no changes in the V_oc_ and FF when altering thickness parameters, but in reality there may be changes in these values that would also affect PCE [8]. These changes are more pronounced in subcells with thicker absorber layers, such as narrow E_g_ perovskites and CIGS [30,31]. Device performance simulations are unable to account for every nonideality that occurs in actual devices; however, the general trends revealed by these calculations are useful in informing future experimental work. If such theoretically optimized devices were fabricated, their simulated performance parameters could be compared with experimental results, and device layer thicknesses and material complex optical properties could be obtained from a method such as spectroscopic ellipsometry to further corroborate or enhance the accuracy of simulations [9].

## 4. Conclusions

Optical models of complete superstrate and substrate tandem solar cells have been developed to generate practical models of previously published EQE spectra based on measured complex optical property spectra of component materials for the purpose of further J_sc_ optimization. These models can be used to easily simulate the effects of altering layer thicknesses and adding anti-reflective layers and evaluation of losses in current solar cell designs. These models also enable us to study the effects of using alternative materials to replace various layers of the device as long as the complex optical response of those materials have been measured. In this way, models replicating the structure of real high-efficiency devices can be used to evaluate practical performance gains from different device structure optimizations.

The performance of tandem perovskite PV devices such as the ones fabricated by Wen et al. and Jošt et al. can be improved by adjusting absorber layer thicknesses, and EQE simulations are a useful tool for optimizing these thicknesses under different illuminations. When optimizing PV devices for space-based applications, the difference in J_sc_ under AM 0 illumination is an important consideration, as the ideal thickness combination may not be the same as that of an optimized terrestrial device under AM 1.5 illumination.

For the all-perovskite tandem device, by increasing the thicknesses of the wide E_g_ and narrow E_g_ perovskite absorber layers from 350 and 975 nm to 356 and 1200 nm, the J_sc_ may be increased from 15.81 to 15.94 mA/cm^2^ under AM 1.5 illumination. This corresponds to an increase in efficiency from 25.83 to 26.05%. Additionally, adding a 115–118 nm thick MgF_2_ anti-reflective layer may further increase J_sc_ to 16.32 mA/cm^2^, corresponding to an efficiency of 26.67%. Under AM 0, a change in absorber layer thicknesses to 310 and 1200 nm increases the J_sc_ from 18.56 to 19.72 mA/cm^2^, which corresponds to an increase in efficiency from 30.33 to 32.22%. By adding 115 nm MgF_2_, the J_sc_ may be further increased to 20.18 mA/cm^2^, increasing the efficiency to 32.84%.

For the perovskite/CIGS device, by increasing the thicknesses of the perovskite layer from 500 to 615 nm, the J_sc_ may be increased from 18.84 to 19.65 mA/cm^2^ under AM 1.5 illumination. Assuming the same values for Voc and FF as reported by Jošt et al., this means the efficiency would increase from 23.74 to 24.76%. Under AM 0 illumination, an increase in the perovskite thickness to 512 nm results in an increase in predicted J_sc_ from 23.13 to 23.34 mA/cm^2^. This corresponds to a predicted efficiency increase from 29.15 to 29.41%.

Absorption, reflection, and electronic losses affect the performance of each layer, and these losses serve as a starting point for further optimization. For example, in the all-perovskite devices, there is significant reflection loss despite the simulated anti-reflective coating. Application of different anti-reflective materials, multilayer coatings, or texturing could improve device performance. Additionally, the ITO exhibits the highest parasitic absorption of any layer in the device; altering its thickness or replacing it with another transparent conductor could also improve device performance. The NiO_x_ and Me4-PACz monolayer also exhibits substantial parasitic absorption at short wavelengths which limits the J_sc_ from the wide E_g_ perovskite top subcells and could be a subject of future research. The CIGS/perovskite device exhibits significant electronic losses which could be reduced by improving the quality of the CIGS layer. The IZO layer also exhibits parasitic absorption at short wavelengths, which could be minimized by altering its thickness or replacing it with another transparent conducting oxide. For space-based applications, there are further considerations not accounted for in this methodology, such as high-energy radiation, thermal cycling and micrometeorite impacts [32]. Overall, the models developed for these two tandem device configurations, benchmarked against experimental high-efficiency devices, serve as a framework to investigate other variations in device structure and material characteristics. This helps to establish realistic practical expectations prior to more costly and time-consuming device fabrication processes.

## Figures and Tables

**Table 1:** Calculated J_sc_ as a function of MgF_2_ anti-reflection coating film thickness of wide and narrow E_g_ subcells of an all-perovskite tandem PV device as well as J_sc_ loss to reflection where the wide E_g_ perovskite is 310 nm thick and the narrow E_g_ is 1200 nm under AM 0 illumination. The narrow E_g_ thickness is chosen as this is the thickest perovskite absorber layer thickness in a tandem device seen in the literature, and the wide E_g_ thickness is chosen to produce current matching conditions under AM 0 illumination.

MgF_2_ Thickness (nm)	Wide E_g_ J_sc_ (mA/cm^2^)	Narrow E_g_ J_sc_ (mA/cm^2^)	Reflection Loss J_sc_ (mA/cm^2^)
0	19.73	19.72	8.84
50	20.06	19.86	8.34
75	20.23	20.00	8.00
100	20.25	20.13	7.85
105	20.23	20.15	7.85
110	20.21	20.16	7.85
115	20.18	20.18	7.86
120	20.15	20.19	7.87
125	20.12	20.20	7.90
150	19.97	20.21	8.04

**Table 2:** Calculated J_sc_ as a function of MgF_2_ anti-reflection coating film thickness of wide and narrow E_g_ subcells of an all-perovskite tandem PV device as well as J_sc_ loss to reflection where the wide E_g_ perovskite is 356 nm thick and the narrow E_g_ is 1200 nm under AM 1.5 illumination. The narrow E_g_ thickness is chosen as this is the thickest perovskite absorber layer thickness in a tandem device seen in the literature, and the wide E_g_ thickness is chosen to produce current matching conditions under AM 1.5 illumination.

MgF_2_ Thickness (nm)	Wide E_g_ J_sc_ (mA/cm^2^)	Narrow E_g_ J_sc_ (mA/cm^2^)	Reflection Loss J_sc_ (mA/cm^2^)
0	15.95	15.94	10.00
50	16.20	16.06	9.60
75	16.35	16.17	9.32
100	16.37	16.27	9.18
115	16.33	16.32	9.17
116	16.32	16.32	9.18
117	16.32	16.32	9.18
118	16.32	16.32	9.18
119	16.31	16.33	9.18
125	16.28	16.34	9.20

**Table 3:** Calculated J_sc_ as a function of LiF anti-reflection coating film thickness of perovskite and CIGS subcells in a tandem PV device as well as J_sc_ loss to reflection where the perovskite is 512 nm thick and the CIGS layer is 2500 nm thick under AM 0 illumination. The CIGS layer thickness is chosen to match the literature, and the perovskite thickness is chosen to produce current matching conditions under AM 0 illumination.

LiF Thickness (nm)	Wide E_g_ J_sc_ (mA/cm^2^)	Narrow E_g_ J_sc_ (mA/cm^2^)	Reflection Loss J_sc_ (mA/cm^2^)
0	20.72	22.76	5.18
50	22.39	22.98	2.97
75	23.32	23.2	1.73
95	23.52	23.32	1.43
100	23.49	23.34	1.46
105	23.43	23.34	1.53
110	23.34	23.34	1.63
115	23.24	23.33	1.76
120	23.12	23.30	1.92

**Table 4:** Calculated J_sc_ as a function of LiF anti-reflection coating film thickness of perovskite and CIGS subcells in a tandem PV device as well as J_sc_ loss to reflection where the perovskite is 615 nm thick and the CIGS layer is 2500 nm thick under AM 1.5 illumination. The CIGS layer thickness to match the literature, and the perovskite thickness is chosen to produce current matching conditions under AM 1.5 illumination.

LiF Thickness (nm)	Wide E_g_ J_sc_ (mA/cm^2^)	Narrow E_g_ J_sc_ (mA/cm^2^)	Reflection Loss J_sc_ (mA/cm^2^)
0	17.32	19.54	3.88
50	18.66	19.56	2.39
75	19.49	19.63	1.48
85	19.67	19.65	1.29
90	19.72	19.66	1.25
95	19.74	19.66	1.24
100	19.74	19.66	1.26
105	19.71	19.66	1.31
110	19.66	19.65	1.39
115	19.59	19.21	2.00
125	19.29	19.59	1.74

**Table 5:** Equivalent J_sc_ losses from parasitic absorption in each device component layer of the optimized all-perovskite tandem PV device under AM 0 illumination, as well as contribution from reflectance and electronic losses.

Layer	Equivalent J_sc_ Collection Losses (mA/cm^2^) (300–1125 nm)	Irradiance Losses (mW/cm^2^) (300–1200 nm)
MgF_2_	0.00	0.00
Glass	0.00	0.00
ITO	2.70	4.06
NiO_x_	0.46	0.81
C_60_ (front)	0.23	0.46
SnO_2_	0.14	0.22
Au	0.78	1.06
C_60_ (rear)	0.01	0.01
PEDOT:PSS	0.00	0.00
BCP	0.00	0.00
Cu	0.43	0.52
Reflectance	7.86	13.57
Electronic loss	0.48	1.26

**Table 6:** Equivalent J_sc_ losses from parasitic absorption in each device component layer of the optimized all-perovskite tandem PV device under AM 1.5 illumination, as well as contribution from reflectance and electronic losses.

Layer	Collection Losses (mA/cm^2^) (300–1120 nm)	Irradiance Losses (mW/cm^2^) (300–1200 nm)
MgF_2_	0.00	0.00
Glass	0.00	0.00
ITO	2.48	3.53
NiO_x_	0.45	0.62
C_60_ (front)	0.16	0.31
SnO_2_	0.11	0.18
Au	0.64	0.85
C_60_ (rear)	0.00	0.00
PEDOT:PSS	0.00	0.00
BCP	0.00	0.00
Cu	0.42	0.51
Reflectance	9.18	12.43
Electronic loss	0.35	0.90

**Table 7:** Equivalent J_sc_ losses from parasitic absorption in each device component layer of the optimized CIGS/perovskite tandem PV device under AM 0 illumination, as well as contribution from reflectance and electronic losses.

Layer	Collection Losses (mA/cm^2^) (300–1200 nm)	Irradiance Losses (mW/cm^2^) (300–1200 nm)
LiF (front)	0.00	0.00
IZO	1.87	5.33
SnO_2_	0.34	0.79
LiF (rear)	0.00	0.00
NiO_x_ and monolayer	0.00	0.00
ZnO	0.00	0.00
CdS	0.07	0.11
Mo	1.61	1.77
Reflectance	1.63	4.79
Electronic loss	4.17	5.92

**Table 8:** Equivalent J_sc_ losses from parasitic absorption in each device component layer of the optimized CIGS/perovskite tandem PV device under AM 1.5 illumination, as well as contribution from reflectance and electronic losses.

Layer	Collection Losses (mA/cm^2^) (300–1200 nm)	Irradiance Losses (mW/cm^2^) (300–1200 nm)
LiF (front)	0.00	0.00
IZO	1.26	3.25
SnO_2_	0.27	0.60
LiF (rear)	0.00	0.00
NiO_x_ and monolayer	0.00	0.00
ZnO	0.00	0.00
CdS	0.05	0.08
Mo	1.49	1.63
Reflectance	1.24	3.52
Electronic loss	3.44	4.73

## Data Availability

The original contributions presented in this study are included in the article/Supplementary Materials. Further inquiries can be directed to the corresponding author.

## Supplementary Materials

The following supporting information can be downloaded at: https://www.mdpi.com/article/10.3390/ma19173618/s1. References [33,34,35,36,37,38,39,40,41] are cited in the Supplementary Materials.
