# Supplementary material for: Optimization of Perovskite Tandem Photovoltaic Devices for Terrestrial and Space-Based Applications Using External Quantum Efficiency Simulations
Source: Materials (Basel). 2026 Aug 26;19(17):3618. doi: 10.3390/ma19173618 (PMC13566085; doi:10.3390/ma19173618)
Supplement: Supplementary file 1 [file materials-19-03618-s001.zip › materials-4478718-supplementary.pdf]

Supplemental Information for Optimization of Perovskite Tandem Photovoltaic Devices for Terrestrial and Space-based Applications using External Quantum Efficiency Simulations

Emily Amonette and Nikolas J. Podraza

Ellipsometric spectra of an indium zinc oxide (IZO) thin film on glass are measured over a spectral range of 0.73—5.88 eV and 60° angle of incidence using a single rotating compensator multichannel ellipsometer (M-2000 FI, J.A. Woollam Co.)[1,2]. A least squares regression analysis with an unweighted error function σ is used to fit a parametric model to experimental data and is described by

$$\sigma = \left[\frac{1}{3n-m}\sum_{i=1}^{n}\left[\left(N_i^{mod} - N_i^{exp}\right)^2 + \left(C_i^{mod} - C_i^{exp}\right)^2 + \left(S_i^{mod} - S_i^{exp}\right)^2\right]\right]^{1/2}, \quad \text{(S1)}$$

where n is the number of measured data points, m is the number of model parameters, and the superscripts "mod" and "exp" refer to model-simulated and experimental data[3,4]. N, C, and S refer to ellipsometry data where $N = \cos 2\psi$, $C = \sin 2\psi \cos\Delta$, and $S = \sin 2\psi \sin\Delta$.

The structural model is shown in Figure S1 and consists of a glass substrate, a 20 ± 10 nm thick nucleation layer consisting of 94 ± 8% IZO and void, a 220 ± 10 nm thick bulk IZO layer, and a 6 ± 2 nm thick surface layer consisting of 30 ± 10% IZO and void. The nucleation and surface layers are modeled using Bruggeman effective medium approximations (EMAs) to describe the mixing of materials and is described by

$$f_a\frac{\varepsilon_a - \varepsilon}{\varepsilon_a + 2\varepsilon} + f_b\frac{\varepsilon_b - \varepsilon}{\varepsilon_b + 2\varepsilon} = 0, \quad \text{(S2)}$$

where ε is the dielectric function of the layer, f_a and f_b are the volume fractions of the two constituent materials, and ε_a and ε_b are their respective optical response functions[5,6].

The optical model of IZO consists of two Gaussian oscillators, a Drude expression, and a constant additive term to the real part of the dielectric function denoted as ε_∞[7—9]. Each Gaussian oscillator is described by

$$\varepsilon_{Gaussian}(E) = A_n(\Gamma(Z^-) + \Gamma(Z^+) + i[\exp(-(Z^-)^2) - exp(-(Z^+)^2)]), \quad \text{(S3a)}$$

where A_n is the amplitude, E_n is the center energy of the oscillator, Γ_n is a convergence series that produces a Kramers-Kronig consistent line shape for ε_1, and Z^+ and Z^- are defined by

$$Z^+ = \frac{E + E_n}{\sigma_n} \qquad Z^- = \frac{E - E_n}{\sigma_n}, \tag{S3b}$$

where

$$\sigma_n = \frac{\Gamma_n}{2\sqrt{ln2}}, \tag{S3c}$$

where Γ_n is the broadening[7,8].

The Drude expression describes the effect of free carriers on the dielectric function by

$$\varepsilon_{Drude}(E) = \frac{-\hbar^2}{\varepsilon_0 \rho \ \ (\tau \ \ \cdot E^2 + i\hbar E)}, \tag{S4}$$

where ε_0 is the permittivity of free space, ρ is resistivity, and τ is mean scattering time[9].

Model parameters are shown in Table S-I, the parametric model fit to ellipsometry data is shown in Figure S2, and IZO dielectric function is shown in Figure S3.

References

[1]Woollam, J.A.; Johs, B.D.; Herzinger, C.M.; Hilfiker, J.N.; Synowicki, R.A.; and Bungay, C.L.; "Overview of variable-angle spectroscopic ellipsometry (VASE): I. Basic theory and typical applications." In Optical Metrology: A Critical Review, vol. 10294, p. 1029402. International Society for Optics and Photonics. Denver, CO. (1999).

[2]Lee, J.; Rovira, Pl I.; An, I.; and Collins, R.W.; "Rotating-compensator multichannel ellipsometry: Applications for real time Stokes vector spectroscopy of thin film growth." Review of scientific instruments 69, no. 4 1800-1810. (1998).

[3]Johs, B. and Herzinger, C.M.; "Quantifying the accuracy of Ellipsometer Systems." Physica Status Solidi C 5, 1031 (2008).

[4]Alterovitz, S.A. and Johs, B.; "Multiple minima in the ellipsometric error function." Thin Solid Films 313–314, 124 (1998).

[5]Aspnes, David E., J. B. Theeten, and F. Hottier. "Investigation of effective-medium models of microscopic surface roughness by spectroscopic ellipsometry." Physical Review B 20, no. 8 (1979): 3292.

[6]Fujiwara, H., Joohyun Koh, P. I. Rovira, and R. W. Collins. "Assessment of effective-medium theories in the analysis of nucleation and microscopic surface roughness evolution for semiconductor thin films." Physical Review B 61, no. 16 (2000): 10832.

[7]Meneses, D. De Sousa, M. Malki, P. Echegut. Structure and lattice dynamics of binary lead silicate glasses investigated by infrared spectroscopy. J. of Non-Crystalline Solids 352, 8, 769-776. (2006).

[8]Peiponen, K.-E. and E.M. Vartiainen. Kramers Kronig relations in optical data inversion. Phys. Rev. B 44 8301. (1991).

[9]Tiwald, T.E., D.W. Thompson, J.A. Woollam, W. Paulson, and R. Hance, "Application of IR variable angle spectroscopic ellipsometry to the determination of free carrier concentration depth profiles" Thin Solid Films 313—314 661 (1998).

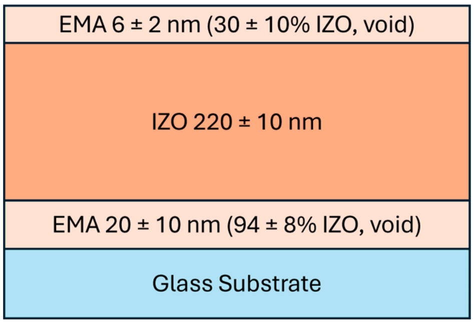

Figure S1. Structural model of bulk indium zinc oxide (IZO) on glass.

Table S1. Model parameters describing the optical response of IZO. $\varepsilon_\infty = 2.40 \pm 0.06$ and $\sigma = 8.723 \times 10^{-3}$.

	A_n	Γ_n (eV)	E_n (eV)	ρ (Ωcm)[2]	τ (fs)[2]
$Gaussian_1$	1.68 ± 0.09	1.36 ± 0.04	4.29 ± 0.02		
$Gaussian_2$	4.04 ± 0.10	3.1 ± 0.2	6.97 ± 0.09		
Drude				$(4.8 \pm 0.2) \times 10^{-4}$	9.9 ± 0.4

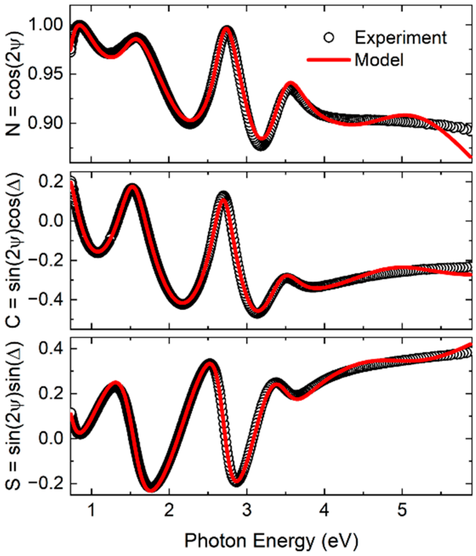

Figure S2. Experimental ellipsometry data (symbols) and model parameterization (lines) in $N = \cos(2\psi)$, $C = \sin(2\psi)\cos(\Delta)$, and $S = \sin(2\psi)\sin(\Delta)$.

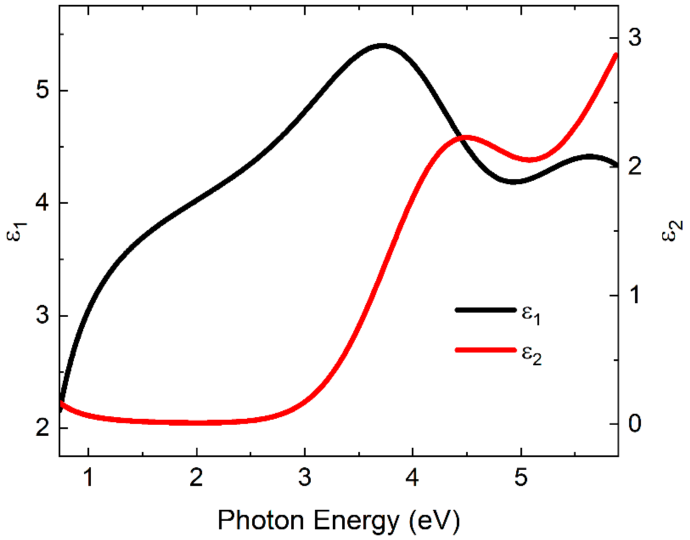

Figure S3. Complex dielectric function ($\varepsilon = \varepsilon_1 + i\varepsilon_2$) spectra of IZO determined via spectroscopic ellipsometry.
